# Early Measles Vaccination During an Outbreak in the Netherlands: Short-Term and Long-Term Decreases in Antibody Responses Among Children Vaccinated Before 12 Months of Age

**DOI:** 10.1093/infdis/jiz159

**Published:** 2019-04-11

**Authors:** Iris D Brinkman, Jelle de Wit, Gaby P Smits, Hinke I ten Hulscher, Maria C Jongerius, Taymara C Abreu, Fiona R M van der Klis, Susan J M Hahné, Marion P G Koopmans, Nynke Y Rots, Debbie van Baarle, Robert S van Binnendijk

**Affiliations:** 1 Center for Infectious Disease Control, National Institute for Public Health and the Environment, Bilthoven; 2 Department of Viroscience, Erasmus Medical Center, Rotterdam; 3 Laboratory of Translational Immunology, Department of Immunology, University Medical Center Utrecht, Utrecht University, Utrecht, the Netherlands

**Keywords:** Antibody response, timing of vaccination, protection, antibody avidity

## Abstract

**Background:**

The majority of infants will not be protected by maternal antibodies until their first measles vaccination, between 12 and 15 months of age. This provides incentive to reduce the age at measles vaccination, but immunological consequences are insufficiently understood, and long-term effects are largely unknown.

**Methods:**

A total of 79 infants who received early measles vaccination between 6 and 12 months age and a second dose at 14 months of age were compared to 44 children in a control group who received 1 dose at 14 months of age. Measles virus–specific neutralizing antibody concentrations and avidity were determined up to 4 years of age.

**Results:**

Infants who first received measles vaccination before 12 months of age had a long-term decrease in the concentration and avidity of measles virus–specific neutralizing antibodies, compared with infants in the control group. For 11.1% of children with a first dose before 9 months of age, antibody levels at 4 years of age had dropped below the cutoff for clinical protection.

**Conclusions:**

Early measles vaccination provides immediate protection in the majority of infants but yields a long-term decrease in neutralizing antibody responses, compared to vaccination at a later age. Additional vaccination at 14 months of age does not improve this. Over the long term, this may result in an increasing number of children susceptible to measles.

## Teaser

Measles vaccination before 9 months of age results in reduced levels of measles virus neutralizing antibodies. Additional vaccination only leads to a short-term increase in antibody levels. Among children who received early vaccination, the number with nonprotective antibody levels increased over the long term.

Since the introduction of measles vaccination, the annual number of measles cases worldwide has notably dropped, with <10 million cases in 2016 [1]. Still, the number of annual measles deaths was estimated to be 109 638 in 2017 [2]. Recent reports show an increase in the number of measles cases in several regions, with a record amount of cases in Europe in 2018 [1, 3–5]. Here, a large proportion of measles cases (range, 15.9%–32.0%) was reported in infants <1 year of age [6–8], a group at high risk for the development of measles-related complications and mortality [9, 10].

At birth, infants are protected from measles by neutralizing maternal antibodies, the levels of which decline over time [11–14]. These antibodies interfere with vaccine responses, which is a major reason to postpone vaccination until after the first year of life [15, 16]. Importantly, recent studies showed that measles virus–specific maternal antibodies from vaccinated mothers only protect infants until 3–4 months of age and are no longer detectable in the majority of infants at 6 months of age [12, 14]. Despite this, vaccination at 6 months of age as compared to 12 or 15 months of age results in lower seroconversion rates and lower levels of neutralizing antibodies shortly after vaccination [16, 17]. Although the early loss of maternal antibodies is a major incentive to reduce the age at first measles vaccination, especially in outbreak settings, long-term effects on measles virus–specific immunity and protection are largely unknown.

During the latest measles epidemic in the Netherlands, infants between 6 and 12 months of age living in areas with a vaccination coverage <90% were offered an additional measles vaccination prior to regular vaccination at 14 months [18]. This provided the opportunity to perform a clinical study to investigate the humoral immune response over the short and long term in relation to the timing of the first measles vaccination.

## METHODS

### Study Population

**Figure 1:**
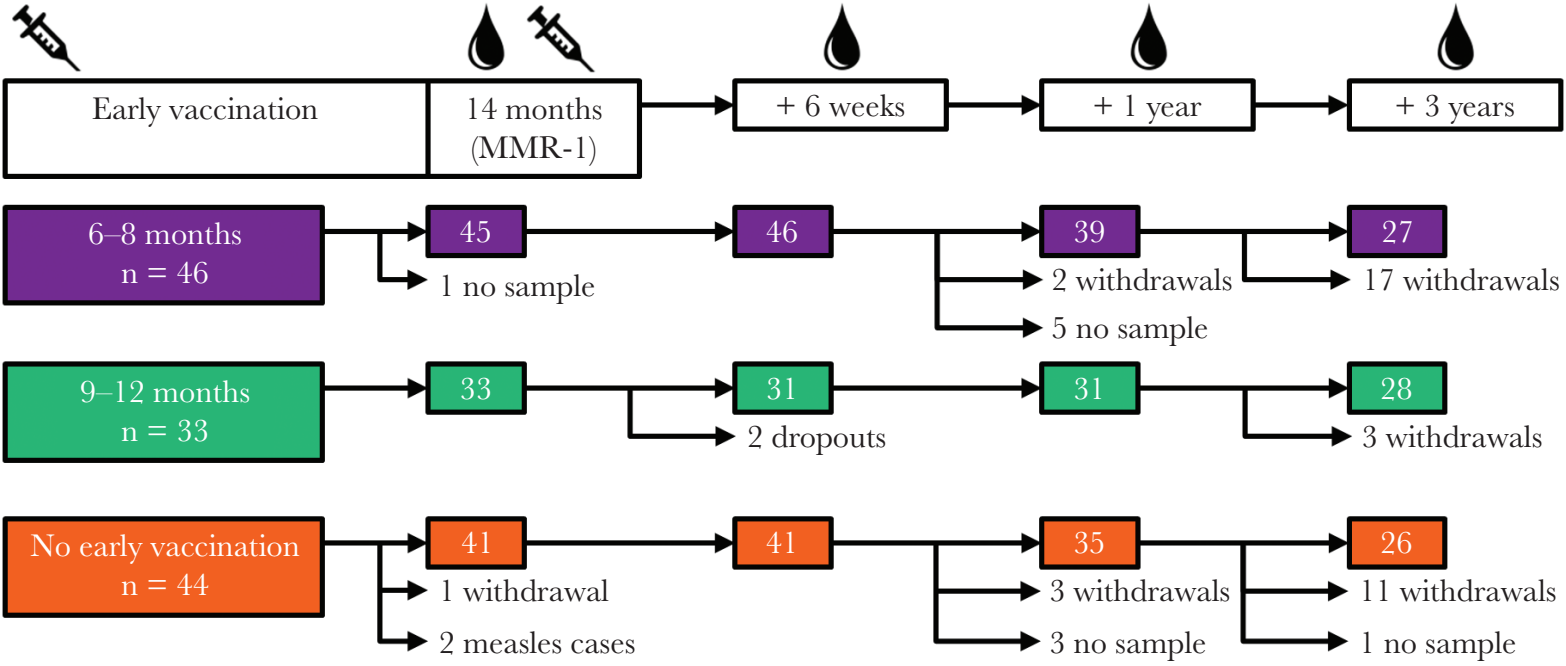
Study design. Children were grouped on the basis of age at first receipt of measles, mumps, and rubella (MMR) vaccine: 6–8 months, 9–12 months, or 14 months. Ages at which blood samples were collected are indicated, as are the number of withdrawals recorded at each home visit. Numbers in colored boxes indicate the number of samples analyzed for each group at each time point. MMR-1, MMR dose received at age 14 months.

During a measles epidemic in the Netherlands from May 2013 until March 2014, parents were invited to enroll their infants in a prospective observational cohort study from week 35 of 2013 up to week 8 of 2014 [19]. In the online baseline questionnaire of this study, parents could indicate whether they could be approached for future research. Parents who agreed to this received an invitation to join this controlled, open-label, parallel-group trial. A total of 79 healthy infants at the time of inclusion who received a first dose of measles, mumps, and rubella vaccine (MMR) between 6 and 12 months of age and a second dose at 14 months of age (hereafter, “MMR-1” denotes MMR received at age 14 months) were included. A control group of 44 infants who received only MMR-1 was selected from the Dutch community-based administration. Informed consent was obtained from parents. Blood specimens were collected through heel/finger prick or venipuncture during home visits at 14 months of age, before MMR-1 receipt, and then 6 weeks, 1 year, and 3 years later (Figure 1). Parents filled out a questionnaire. Exclusion criteria were receipt of immunosuppressive medication, presence of a known or suspected immunological or bleeding disorder, and assumed previous measles virus infection (based on positive results of serologic testing), which is why 2 children were excluded from the control group. The study was approved by the Medical Research Ethics Committees United (METC Noord-Holland, Alkmaar, the Netherlands; clinical trials registration NL45616.094.13).

### Measles Vaccination

Before enrollment, infants had already received MMR-VaxPro vaccine (RVG 17672; Sanofi Pasteur-MSD), containing live attenuated measles virus (Enders’ Edmonston strain), mumps virus (Jeryl Lynn [level B] strain), and rubella virus (Wistar RA 27/3 strain), between 6 and 12 months of age. The same vaccine was administered to all children at 14 months of age during a home visit.

### Laboratory Analysis

Serum samples were obtained from whole-blood specimens and stored at −20°C until use. Measles virus–specific neutralizing antibodies were measured in a modified plaque reduction neutralization test endorsed by the World Health Organization (WHO) [20]. Two-step serum dilution series were made in triplicate in 96-well culture plates. The WHO 3rd International Standard for measles virus antibody (3 IU/mL; NIBSC code 97/648) was included. Incubation with laboratory-adapted Edmonston virus and Vero cells was performed according to a previously established protocol [21, 22]. Measles virus–infected foci were stained after 48 hours with anti-measles virus nucleoprotein antibody (clone 83KKII; Merck Millipore). Measles virus–specific neutralizing antibody concentrations are expressed as IU/milliliter, based on the 50% plaque reduction dilution of the serum and standard. A concentration of ≥0.12 IU/mL was considered to be protective [21, 23].

Measles virus–specific immunoglobulin G (IgG) avidity was determined by modifying an established bead-based multiplex immunoassay for measles virus serum IgG [24]. Briefly, after incubation of measles virus–conjugated fluorescent beads with serum and before staining, beads were washed with phosphate-buffered saline and subsequently left untreated or treated for 10 minutes with 1.5 M ammonium thiocyanate (NH_4_SCN; Merck Millipore). This condition showed a separation between low- and high- avidity samples comparable to that in previously published assays [25]. The avidity index is defined as the percentage of antibodies that remain bound to the beads after treatment with NH_4_SCN, calculated as: [amount of IgG after NH_4_SCN treatment]/[amount of IgG without NH_4_SCN treatment] × 100.

### Statistical Analysis

Statistical analyses were performed using GraphPad Prism (version 7.03), R package 3.4.4, and IBM SPSS statistics (version 24). The geometric mean measles virus–specific neutralizing antibody concentration (GMC) and geometric mean avidity index were calculated. For statistical analysis, individual measles virus–specific neutralizing antibody concentrations were log_10_ transformed. Children were grouped on the basis of their age at first receipt of the vaccine. Differences in the percentage of protected children were calculated using the Fisher exact test. Differences between GMCs of groups were tested by the Kruskal-Wallis test and corrected for multiple comparisons by using the Dunn test. The Friedman test was performed to analyze differences over time. Children with missing data from ≥1 time point were excluded.

We fitted a linear model to log_10_-transformed antibody levels, which gives a constant fraction of antibodies disappearing over time, assuming that the antibody decay rate per individual was constant. The exponential decay rate was determined by fitting a linear mixed-effects random intercept regression model [26] on log(neutralizing antibody concentrations) for all children 6 weeks, 1 year, and 3 years after receipt of MMR-1. Early vaccinated children with antibody levels of ≤0.12 IU/mL at 14 months of age were excluded. The model accounts for differences in the antibody concentration (the random intercept) between infants. Age groups, time after MMR-1 receipt, and an interaction term between these variables were added as fixed effects in the model to account for a possible difference in the rate of exponential decay by age group.

*P* values ≤.05 indicate statistically significant differences.

## RESULTS

### Study Characteristics

In total, 79 children who received a first vaccine dose between ages 6 and 12 months and a second dose (ie, MMR-1) at age 14 months were included in the study, together with a control group of 44 children who received only MMR-1. Three years after MMR-1 receipt, parents were approached again for inclusion, which resulted in a relatively large withdrawal proportion of 25% (23 of 113 children; Figure 1). Group sizes were rather similar at this time point, and neutralizing antibody GMCs were comparable at all time points between the full cohort and children who completed the study at 4 years of age (data not shown). This implies that the children who withdrew from the study created no bias.

Baseline characteristics of early vaccinated children and the control group are shown in Table 1. All groups were comparable with respect to period of birth, sex, birth year of mothers, duration of pregnancy, and percentage of mothers breastfeeding their children, except that, among mothers of infants in the early vaccinated groups, a greater proportion received measles vaccine (*P* = .001) but a smaller proportion had measles (*P* = .045) during childhood, compared with mothers of infants in the control group. Although we have no explanation for these differences, we assume that the higher percentage of children born to naturally infected mothers in the control group did not influence our results, because maternal antibodies are not expected to be present [14] and were not detected at 14 months of age.

**Table 1:** Baseline Characteristics

Characteristic	Age at Primary Vaccination			*P*
	6–8 mo (n = 44)	9–12 mo (n = 31)	14 mo (Control) (n = 40)	
Birth date	21 Dec 2012 (2 Nov 2012–25 Jul 2013)	2 Sep 2012 (2 Aug 2012–27 Sep 2012)	15 Jun 2013 (8 Aug 2012–23 Aug 2013)	
Male sex	19 (43.2)	15 (48.4)	17 (42.5)	.867^a^
Birth year of mother	1982 (1971–1989]	1982 (1967–1989)	1982 (1971–1988)	.545^b^
Pregnancy duration, wk^a^	39.5 (31–42]	39 (29–42)	40 (36–44)	.310^b^
Breastfeeding^b^				
Yes	24 (54.5)	13 (41.9)	18 (45.0)	.508^a^
No	20 (45.5)	18 (58.0)	22 (55.0)	
Childhood measles in mother				
Yes	5 (11.4)	5 (15.2)	14 (35.0)	.045^a^
No	29 (65.9)	19 (61.3)	23 (57.5)	
Unknown	10 (22.7)	7 (22.6)	3 (7.5)	
Measles vaccine receipt by mother				
Yes	38 (86.4)	25 (80.6)	26 (65)	.001^a^
No	2 (4.5)	2 (6.5)	13 (32.5)	
Unknown	4 (9.1)	4 (12.9)	1 (2.5)	

Data are median (range) or no. (%) of infants.

^a^By the χ^2^ test.

^b^By the Kruskal-Wallis test.

### MMR Receipt Before 9 Months of Age Resulted in Decreased Neutralizing Antibody Concentrations

Measles virus–specific neutralizing antibody concentrations were determined at 14 months of age and 6 weeks, 1 year, and 3 years later (Supplementary Table 1). Measles virus–specific neutralizing antibody concentrations relative to age (in days) at receipt of MMR are shown for each individual child in Supplementary Figure 1*A*–*D*.

**Figure 2:**
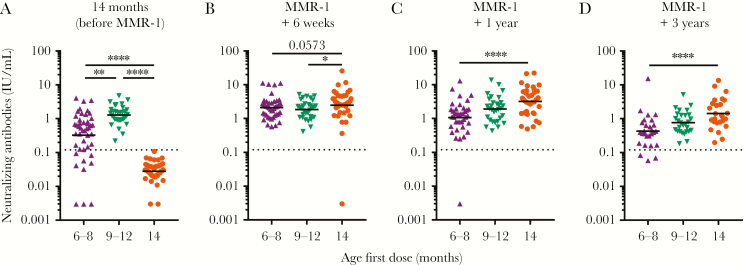
Measles virus–specific neutralizing antibodies measured by a focus reduction neutralization test (FRNT) in children who received their first measles, mumps, and rubella vaccine (MMR) dose at 6–8 months, 9–12 months, or 14 months of age. Differences in geometric mean concentrations between groups, represented as horizontal bars, were compared by the Kruskal-Wallis test at 14 months of age (*A*) and then 6 weeks (*B*), 1 year (*C*), and 3 years later (*D*). The dashed gray line indicates the level of antibody sufficient for protection from clinical measles (ie, 0.12 IU/mL). Two controls assumed to have measles, based on positive serologic test results at 14 months of age, were excluded from analysis. MMR-1, MMR dose received at age 14 months. *P ≤ .05, **P ≤ .01, and ****P ≤ .0001.

Group-wise analysis revealed that, before receipt of MMR-1, children who received their first dose between 6 and 8 months of age had lower measles virus–specific neutralizing antibody GMCs than children who received their first dose between 9 and 12 months of age (*P* = .002; Figure 2A). All children vaccinated between 9 and 12 months of age had antibody concentrations above the cutoff for clinical protection, whereas 20% of children (9 of 45) vaccinated between ages 6 and 8 months did not (*P* = .001).

Six weeks after MMR-1 receipt, measles virus–specific neutralizing antibodies were detected in all children from the control group except 1 (2.5%), which can be considered a primary vaccination failure (Figure 2B). Unfortunately, no follow-up samples were available because this child withdrew from the study. At this time, GMCs were higher in the control group as compared to those in children with a first dose between ages 6 and 8 months (*P* = .057) and 9–12 months (*P* = .025). All early vaccinated children had antibody levels above the cutoff for protection.

One and 3 years after receipt of their last vaccine dose, a downward trend was observed in neutralizing antibody concentrations, which was strongest for children who received a first dose between ages 6 and 8 months. While the differences at these later time points between the control group and children first vaccinated between 9 and 12 months of age were not significant (*P* = .13 for comparisons at each time point), children who received the first dose between ages 6 and 8 months had clearly lower GMCs (*P* < .0001; Figure 2C and 2D). No distinction could be made between early vaccinated children who received their first dose between ages 6 and 8 months and 9 and 12 months 1 year (*P* = .14) and 3 years after MMR-1 (*P* = .08). All children with a first dose between ages 9 and 12 months and the control group had measles virus–specific neutralizing antibodies that were still above the cutoff for protection 3 years after receipt of their last dose. One year after MMR-1 receipt, 1 child (2.6%) who received a first dose between 6 and 8 months of age had measles virus–specific neutralizing antibodies below the cutoff for protection, which increased to 3 children (11.1%) 3 years after MMR-1 receipt (*P* = .111 for the comparison between early vaccine recipients aged 6–8 months and the control group). For all groups and time points analyzed, no sex-based differences were observed (data not shown).

### MMR Receipt Before 12 Months of Age Resulted in Lower Antibody Avidity

**Figure 3:**
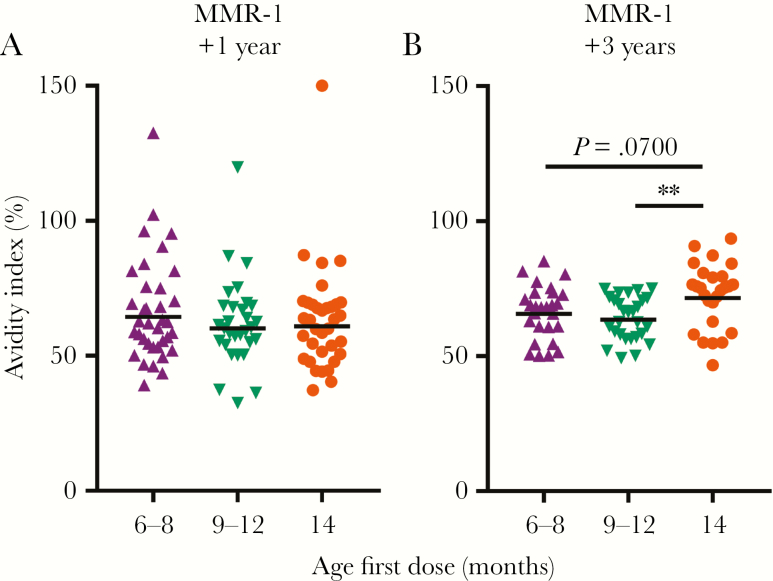
Avidity of measles virus–specific immunoglobulin G (IgG) in children who received their first measles, mumps, and rubella vaccine (MMR) dose at 6–8 months, 9–12 months, or 14 months of age. Avidity was measured 1 and 3 years after receipt of vaccine at 14 months of age. Horizontal bars represent geometric mean values, which were compared by the Kruskal-Wallis test. MMR-1, MMR dose received at age 14 months. **P ≤ .01.

Differences in neutralizing antibody concentrations between groups increased over time, which may indicate differences in antibody avidity maturation. We compared antibody avidity 1 and 3 years after receipt of the last vaccine dose, to represent time points of more complete avidity maturation [27] (Figure 3). After 1 year, we did not observe any differences (Figure 3A), but after 3 years, early vaccinated children had antibodies of lower avidity than children with a first dose at 14 months of age (*P* = .07 and *P* = .005 for comparisons between the control group and infants with the first dose at ages 6–8 months and 9–12 months, respectively; Figure 3B). We did not observe a correlation between neutralizing antibody concentrations and avidity index (data not shown).

### MMR-1 Receipt Only Temporarily Increased Antibody Concentrations in Children First Vaccinated Before 9 Months of Age

**Figure 4:**
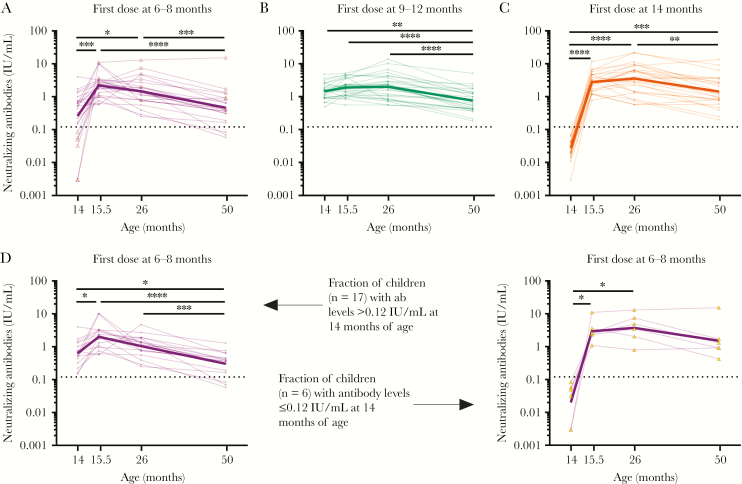
*A*–*C*, Individual measles virus–specific neutralizing antibody concentrations over time for children who first received measles, mumps, and rubella vaccine (MMR) between ages 6 and 8 months (*A*), 9 and 12 months (*B*), and 14 months (*C*) of age. *D*, Children who received their first MMR dose between 6 and 8 months of age were divided into children without and those with nonprotective antibody levels at 14 months of age. Neutralizing antibody concentrations were determined before vaccine receipt at 14 months of age and then 6 weeks, 1 year, and 3 years later and then compared by the Friedman test. The bold lines represent geometric mean concentrations for each group. *P ≤ .05, **P ≤ .01, ***P ≤ .001, and ****P ≤ .0001.

Next, we studied individual longitudinal dynamics and observed different response patterns between the groups (Figure 4). Children first vaccinated between 6 and 8 months of age showed an increased neutralizing antibody titer within 6 weeks after the second dose of measles vaccine (Figure 4A). However, antibody levels significantly dropped in the subsequent 3 years, some of which decreased to levels observed before receipt of MMR-1. This became more apparent when the 6 children who had no protective antibody levels before receipt of MMR-1 were excluded from analysis (Figure 4D). These 6 children showed a strong increase in neutralizing antibody levels after MMR-1 receipt, comparable to the control group (Figure 4C and 4D). Antibody levels in children who received their first dose between 9 and 12 months of age increased only slightly after receipt of MMR-1 and declined in the subsequent 3 years (Figure 4B).

**Figure 5:**
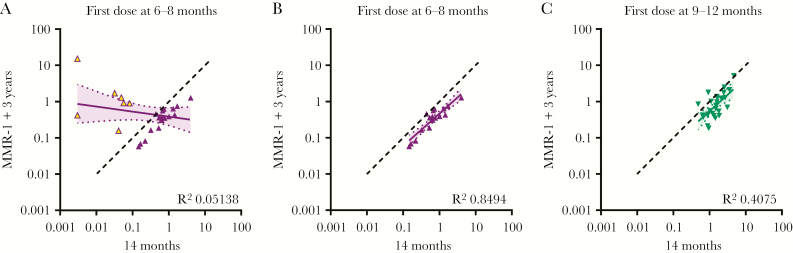
Correlations calculated by linear regression between antibody concentrations determined prior to receipt of a measles, mumps, and rubella vaccine (MMR) dose at 14 months of age (x-axis) and 3 years later (y-axis). (A) All children with a first MMR dose between 6 and 8 months of age, (B) selection of children with a first MMR dose between 6 and 8 months of age and protective antibody levels at 14 months of age (C), and children with a first MMR dose between ages 9 and 12 months are shown.

The antibody responses after vaccination between 6 and 8 months were predictive for the long-term responses following receipt of MMR-1, but only for children who had responded to the first dose (Figure 5A and 5B). This correlation was not as strong for children who received their first dose between 9 and 12 months of age (Figure 5C).

### Antibody Concentrations Declined Faster After Early Vaccination

**Figure 6:**
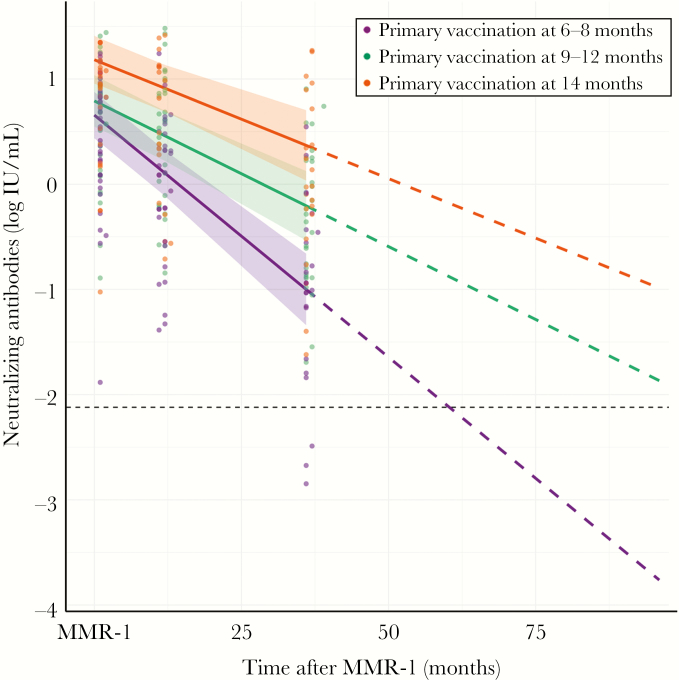
Individual observed log measles virus–specific neutralizing antibody concentrations versus time after receipt of the first dose of measles, mumps, and rubella vaccine. Fitted lines per age group were obtained with a linear mixed-effects regression model. The horizontal dashed line indicates the level of antibody sufficient for protection from clinical measles (ie, 0.12 IU/mL). Dashed lines indicate projection of log antibody levels over time up to 9 years of age, assuming monophasic decay. Shaded areas indicate 95% confidence intervals. Neutralizing antibody concentrations at 14 months of age were excluded. MMR-1, MMR dose received at age 14 months.

Because of the individual variability in the decline of antibody levels following measles vaccination, we applied mixed modeling to calculate the exponential decay of the antibody response until 9 years of age (Figure 6). At this time point, Dutch children normally receive their second measles vaccination. For children who received a first dose between 9 and 12 months of age, antibodies decreased at a faster rate per month (log value, −0.01) than those of the control group, but this difference was not significant (Supplementary Table 2). Based on this model, antibody levels were also expected to remain above the threshold for protection until the next vaccination. However, children who received a first dose between 6 and 8 months of age showed a faster decay of their antibodies per month (log value, −0.03; *P* < .01) as compared to the control group, and the model predicted that, 5 years after MMR-1 receipt, antibodies will have dropped below the threshold for protection.

## DISCUSSION

In this study, we showed that most children develop protective measles virus–specific antibodies following vaccination as early as 6 months of age. A lower risk of measles virus infection was indeed shown for infants first vaccinated between 6 and 14 months of age during this measles outbreak [19]. Because low numbers of measles cases were reported, vaccine effectiveness could not accurately be determined; however, all reported measles cases occurred in infants vaccinated before 9 months of age [19]. We found that 20% infants vaccinated before 9 months of age had measles virus–specific neutralizing antibody levels below the cutoff for clinical protection (0.12 IU/mL), which highlights that a proportion of these infants is not sufficiently protected after 1 dose of MMR. It should be noted that the interval between early vaccination and antibody level measurement at age 14 months increased with a lower age at receipt of the first dose, which may account for some of the quantitative differences between the 2 early vaccinated groups.

Interestingly, for the subgroup of children with low levels of neutralizing antibodies after receipt of the first MMR dose, the response to MMR-1 was quite similar to that for the control group, indicating a primary antibody response after a receipt of MMR-1. This finding was substantiated by the observation that, at 4 years of age, all children had antibody levels above the cutoff for protection. Gans et al also showed that additional vaccination 12 months after primary vaccine failure at 6 months of age resulted in protective antibody levels 2 years later [16]. These results stress that additional vaccination is essential to protect infants who do not respond to a first dose received before 9 months of age.

Previous studies have shown that vaccination before 9 months of age induces a reduced antibody response [16, 17]. Recently, Carazo Perez et al showed that measles virus–specific vaccine response rates increase with increasing age at first vaccination, an effect that persists after additional vaccination [28]. However, another study showed a comparable response to a first dose of MMR received at 8 versus 12 months of age, based on measles virus–specific antibodies measured by enzyme-linked immunosorbent assay [29].

We showed an increased decline in the neutralizing antibody concentration after additional vaccination in early vaccinated children as compared to the control group. This was clearly observed 3 years after vaccination, when antibody concentrations for 11.1% of children were found to have dropped below the cutoff for clinical protection. Previous studies show conflicting results about long-term protection after early vaccination [30, 31], which may be partially explained by the use of different vaccines and different study designs and populations. However, long-term effects are of specific concern, as a gradual decrease in neutralizing antibody concentrations may result in increasing numbers of unprotected individuals in the population over the long term.

MMR-1 receipt seems to have had little effect in children who received their first dose between 9 and 12 months of age. Here, the time between the first and second dose may have been critical; hence, we cannot exclude the possibility that postponing the second vaccination to later time points may result in increased antibody levels.

It should be noted that the effects of first vaccination between ages 9 and 12 months were still not similar to those of first vaccination at 14 months of age. Children first vaccinated at 14 months of age had approximately twice the amount of measles virus–specific neutralizing antibodies, but this difference was not statistically significant, possibly because of the relatively small sample size. Furthermore, antibody avidity was significantly higher for children from the control group at 4 years of age, even though this did not reach statistical significance for children with a first dose between 6 and 8 months of age. This may be caused by the relatively small differences in avidity indexes between the groups and by the small sample size. Nair et al showed no differences between antibody avidity with respect to the age at first vaccination [32], although avidity was measured only 6 months after the last vaccination. Similarly, we did not find any differences in avidity up to 1 year after vaccination between the early vaccinated groups and controls, indicating that this period is too short to measure differences in antibody avidity. Together, our results indicate that differences between groups become larger over time. Even though the majority of children are still protected 3 years after their last vaccination, this may not be the case at a later age.

The reduced vaccine response in the early vaccinated group, especially for the 9 children who showed a low response at 14 months of age, may be explained by the presence of maternal antibodies at the time of vaccination. Unfortunately, we were unable to investigate this because children were included after early vaccination, to avoid interference with the decision to vaccinate. However, a large cross-sectional serologic study performed in the Netherlands in 2006 showed that, on average, infants had maternal antibody levels below the protective level at 3.3 months of age, so we did not expect maternal antibodies to be present after 6 months of age [14]. In addition, the majority of mothers of early vaccinated infants were vaccinated during childhood, resulting in lower levels of maternal antibodies in their infants [19]. For these reasons, we expect that the influence of maternal antibodies on vaccination responses after 6 months was negligible.

Gans et al showed that children first vaccinated at 6 months of age in the absence of maternal antibodies had a reduced antibody response, compared with children first vaccinated at 9 or 12 months of age [16]. This suggests a negative influence of the developing infant immune system on the vaccine response after early vaccination [33]. It is known that infants have fewer and less functional dendritic cells [34], and the number of dendritic cells has been positively correlated with the measles virus–specific antibody response [35]. Second, the neonatal immune system is generally thought to be skewed toward a T-helper type 2 response, because fewer T-helper type 1 cytokines are produced [36]. As a consequence, reduced dendritic cell priming and an altered T-cell response may result in suboptimal B-cell activation. This is supported by the observations that antibody responses changed qualitatively and quantitatively over time.

Several studies have shown a nonspecific beneficial effect on infant mortality after measles vaccination, as reviewed by Higgins et al [37]. We do not have information about morbidity or mortality in our cohort. Based on the small cohort size and the low childhood mortality rate in the Netherlands, we do not expect to be able to measure any nonspecific beneficial effects of early measles vaccination in this setting. However, for future decisions about the timing of measles vaccination, these nonspecific beneficial effects should be taken into consideration.

In conclusion, the majority of children who received early MMR vaccination still had protective antibody levels at 14 months of age. This is expected to protect infants in an outbreak setting. However, reducing the age at first measles vaccination should be carefully assessed on an individual basis, owing to concerns about long-term protection against measles: because the susceptibility to measles virus infection among early vaccinated individuals may increase with age, herd immunity in the population might be adversely impacted. This notion is supported by the observations that, in a proportion of children first vaccinated at <9 months of age, antibody levels had already dropped below the cutoff of clinical protection at 4 years of age even though the children had been vaccinated twice. Reduced protection in early vaccinated individuals may contribute to an already decreasing level of vaccination coverage and increase the risk of future epidemics.

## Supplementary Data

Supplementary materials are available at *The Journal of Infectious Diseases* online. Consisting of data provided by the authors to benefit the reader, the posted materials are not copyedited and are the sole responsibility of the authors, so questions or comments should be addressed to the corresponding author.

jiz159_suppl_Supplementary_Figure_1

jiz159_suppl_Supplementary_Figure_Legends

jiz159_suppl_Supplementary_Table_1

jiz159_suppl_Supplementary_Table_2

## Notes

***Acknowledgments***. We thank Hendriek Boshuizen and Hester Korthals Altes, for their input on statistical modeling; and Nikita Ramsoebhag, for her contribution to the antibody avidity assay. I. B., J. d. W., S. H., D. v. B., N. R., and R. v. B. provided input during writing of the manuscript. I. B., G. S., and H. t. H. were responsible for laboratory analysis. T. C. A. was responsible for modeling the data. R. J., N. R., F. v. d. K., and R. v. B. designed the study. The first draft was written by I. B. All authors contributed to the final version of the manuscript.

***Financial support.*** This work was supported by the Dutch Ministry of Health, Welfare, and Sport.

***Potential conflicts of interest.*** All authors: No reported conflicts of interest. All authors have submitted the ICMJE Form for Disclosure of Potential Conflicts of Interest. Conflicts that the editors consider relevant to the content of the manuscript have been disclosed.
