# Supplementary figures and images for: Early Measles Vaccination During an Outbreak in the Netherlands: Short-Term and Long-Term Decreases in Antibody Responses Among Children Vaccinated Before 12 Months of Age
Source: J Infect Dis. 2019 Apr 11;220(4):594–602. doi: 10.1093/infdis/jiz159 (PMC6639599; doi:10.1093/infdis/jiz159)

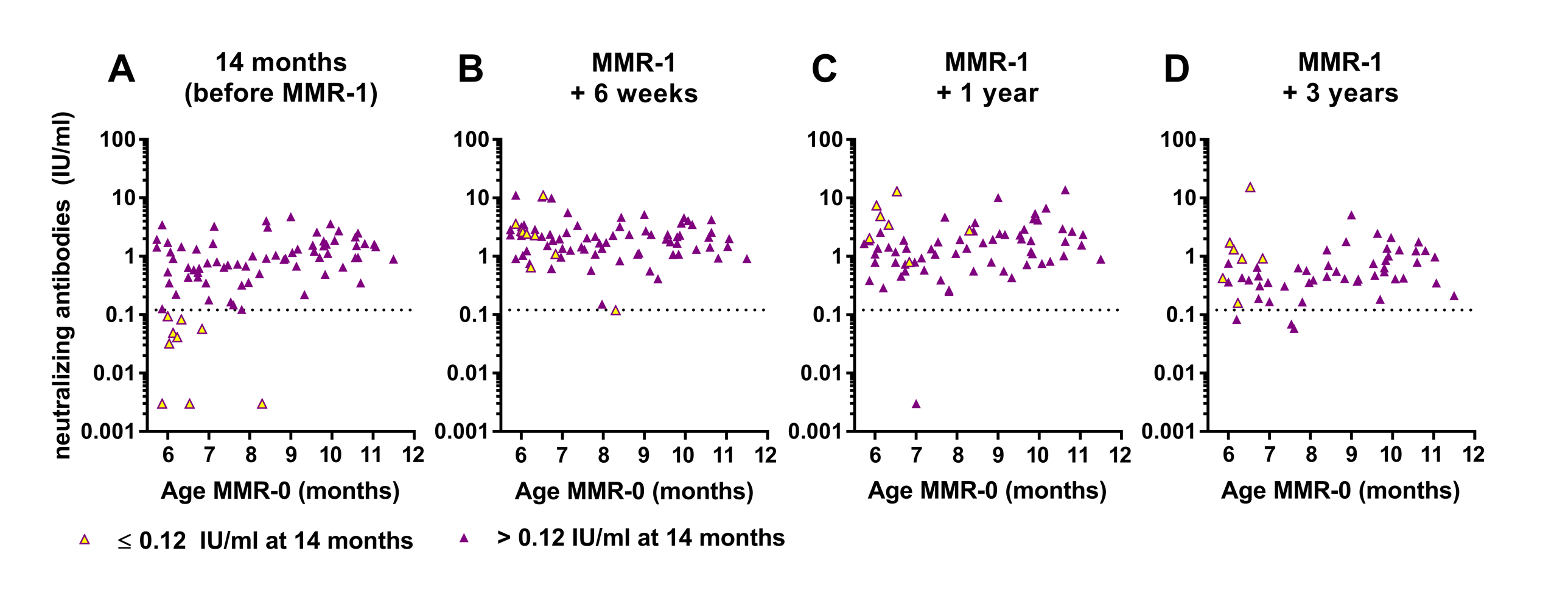

Supplement: jiz159_suppl_Supplementary_Figure_1 [file jiz159_suppl_supplementary_figure_1.png]
