# Supplementary material for: Early Measles Vaccination During an Outbreak in the Netherlands: Short-Term and Long-Term Decreases in Antibody Responses Among Children Vaccinated Before 12 Months of Age
Source: J Infect Dis. 2019 Apr 11;220(4):594–602. doi: 10.1093/infdis/jiz159 (PMC6639599; doi:10.1093/infdis/jiz159)
Supplement: jiz159_suppl_Supplementary_Figure_Legends [file jiz159_suppl_supplementary_figure_legends.docx]

**Supplementary figure 1** Measles neutralizing antibodies based on age of first MMR vaccination at 14 months of age (A), 6 weeks (B), 1 year (C) and 3 years later (D). Dotted horizontal line represent the cutoff for protective neutralizing antibody levels (≥0.12 IU/ml).
