# Supplementary material for: Early Measles Vaccination During an Outbreak in the Netherlands: Short-Term and Long-Term Decreases in Antibody Responses Among Children Vaccinated Before 12 Months of Age
Source: J Infect Dis. 2019 Apr 11;220(4):594–602. doi: 10.1093/infdis/jiz159 (PMC6639599; doi:10.1093/infdis/jiz159)
Supplement: jiz159_suppl_Supplementary_Table_1 [file jiz159_suppl_supplementary_table_1.docx]

|  |  | **6-8 months** | **9-12 months** | **14 months** |
| --- | --- | --- | --- | --- |
| 14 months (before MMR-1) | GMC neutralizing antibodies [95% CI] | 0.33 [0.19-0.55] | 1.28 [1.02-1.61] | 0.03 [0.02-0.04] |
|  | % protective antibody levels [95% CI] | 80% [68.3-91.7%] | 100% | 0% |
| MMR-1 + 6 weeks | GMC neutralizing antibodies [95% CI] | 2.11 [1.70-2.61] | 1.85 [1.46-2.33] | 2.49 [1.65-3.73] |
|  | % protective antibody levels [95% CI] | 100% | 100% | 97.5% [92.7-102.3%] |
| MMR-1  + 1 year | GMC neutralizing antibodies [95% CI] | 1.06 [0.69-1.64] | 1.91 [1.40-2.61] | 3.22 [2.30-4.52] |
|  | % protective antibody levels [95% CI] | 97.4% [92.5-102.4%] | 100% | 100% |
|  | GMC avidity index [95% CI] | 64.4 [58.9-70.3] | 60.2 [54.7-66.2] | 61.0 [55.8-66.7] |
| MMR-1 + 3 years | GMC neutralizing antibodies [95% CI] | 0.43 [0.27-0.67] | 0.76 [0.57-1.03] | 1.41 [0.93-2.15] |
|  | % protective antibody levels [95% CI] | 88.9% [77.0-100.7%] | 100% | 100% |
|  | GMC avidity index [95% CI] | 65.6 [61.8-69.7] | 63.5 [60.4-66.7] | 71.5 [66.6-76.8] |

**Supplementary table 1.** Measles-specific vaccine response before MMR-1 at 14 months, 6 weeks, 1 year and 3 years later, represented by neutralizing antibodies (IU/ml), the percentage of children with neutralizing antibody levels above the protection cutoff (≥0.12 IU/ml) and antibody avidity (AI) for children who received their first vaccination between 6-8 months, 9-12 months and 14 months of age.
