# Supplementary material for: Early Measles Vaccination During an Outbreak in the Netherlands: Short-Term and Long-Term Decreases in Antibody Responses Among Children Vaccinated Before 12 Months of Age
Source: J Infect Dis. 2019 Apr 11;220(4):594–602. doi: 10.1093/infdis/jiz159 (PMC6639599; doi:10.1093/infdis/jiz159)
Supplement: jiz159_suppl_Supplementary_Table_2 [file jiz159_suppl_supplementary_table_2.docx]

|  | **Age first MMR** | **Variable** |
| --- | --- | --- |
| Constant (antibody level at 15.5 months of age) | 6-8 months | 0.66 (0.16)** |
|  | 9-12 months | 0.79 (0.17)* |
|  | 14 months | 1.18 (0.11)** |
| Exponential antibody decay rate (per month) | 6-8 months | -0.05 (0.01)** |
|  | 9-12 months | -0.03 (0.01) |
|  | 14 months | -0.02 (0.004)** |
| Observations |  | 289 |
| Aikaike Inf. Crit. |  | 690.23 |

***Supplementary table 2*** *Model statistics indicating constant and exponential antibody decay rates (per month) of children who received their first MMR at 6-8 months, 9-12 months, and 14 months of age. Constant and exponential decay rate are indicated in log values (standard deviation).*
